# Problems and Solutions in Click Chemistry Applied to Drug Probes

**DOI:** 10.1038/srep35579

**Published:** 2016-10-26

**Authors:** Weilong Zhong, Bo Sun, Cheng Lu, Hengheng Yu, Changhua Wang, Lingfei He, Ju Gu, Shuang Chen, Yanrong Liu, Xiangyan Jing, Zhun Bi, Guang Yang, Honggang Zhou, Tao Sun, Cheng Yang

**Affiliations:** 1 State Key Laboratory of Medicinal Chemical Biology and College of Pharmacy, Nankai University, Tianjin, China; 2 Tianjin Key Laboratory of Molecular Drug Research, Tianjin International Joint Academy of Biomedicine, Tianjin, China

## Abstract

Small-molecule fluorescent probes have been widely used in target identification, but this method has many disadvantages. For example, the identified proteins are usually complex, and additional biochemical studies are needed to distinguish real targets from interference results. To address this problem, we propose a series of strategies for improving the efficiency of target identification. First, pretreatment with a lower concentration of hydrogen peroxide can shield against thiol interference. Second, the use of benzophenone as a photo-affinity group is not appropriate, and diazirines are preferred. Third, if cytoskeleton proteins or stress proteins are captured, the interference must be carefully eliminated. The specificity of target identification can be improved by optimizing these three strategies. In this paper, we discuss the problems associated with the use of the click reaction in living cells and provide important complementary techniques for photo-affinity probes based on the click chemistry reaction.

Small-molecule fluorescent probes, especially affinity-based probes (AfBPs) are essential materials for target identification^1^,^2^. Azide-alkyne “click” reactions have been widely applied to a variety of biomolecules, both *in vitro* and *in vivo*^3^,^4^,^5^. Photo-affinity groups, which include benzophenones and diazirines, are used to increase the binding affinity between drugs and proteins^6^,^7^,^8^,^9^. Much of the research on drug targets has relied on this method^10^. Most drugs bind in the active site of target proteins non-covalently. A chemically “traceable” tag was used to make the original bioactive compounds visible and/or to capture them. The design method must maintain the original form of the protein-ligand interaction that occurs *in situ*. However, a large number of active metal ions or molecules can interfere with photo-crosslinking and the click reaction in living cells and therefore decrease the accuracy of target identification^11^. The use of AfBPs based on the click reaction has been limited. In this study, we systematically analyzed the progress of the click reaction in living cells and identified several problems, including the interference of a thiol residue, the non-specificity of the photo-affinity group, and the interference of captured cytoskeleton proteins or stress proteins. Finally, we propose a series of strategies for improving the efficiency of target identification.

## Results and Discussion

**Figure 1:**
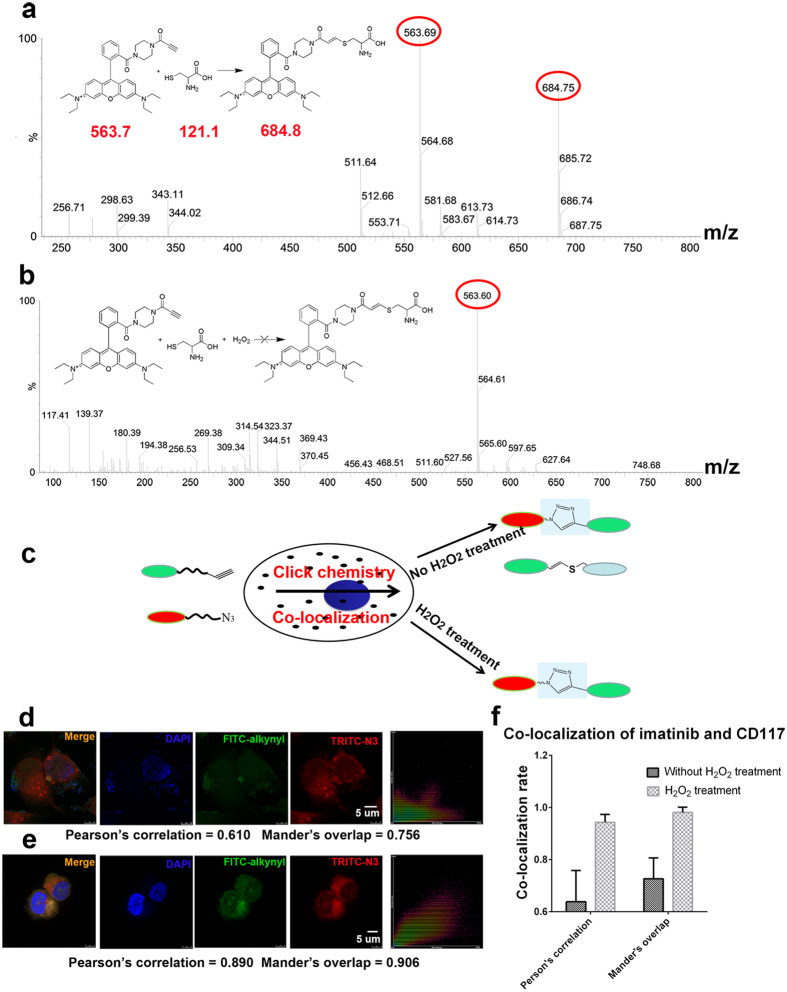
Mass spectrometry analysis of cysteine and alkyne and co-localization of FITC-alkyne and TRITC-azide. (**a**) The interference of cysteine in the probe fishing experiment is shown. (**b**) Treatment with hydrogen peroxide prevents cysteine interference. (**c**) Schematic diagrams depicting the oxidation treatment of experimental cells with hydrogen peroxide. (**d**) The results of the conventional co-localization experiment indicating low co-localization efficiency. (**e**) Co-localization after oxidation treatment with hydrogen peroxide. The degree of co-localization of FITC-alkyne and TRITC-azide is significantly increased. (**f**) Co-localization of imatinib and CD117; the co-localization rate increased after treatment with hydrogen peroxide.

It is generally understood that organic azides efficiently react with alkynes to form 1,2,3-triazole in cells undergoing copper catalysis^5^. However, the reaction is not efficiently completed in cells because of the presence of a large number of interfering molecules and free catalytic ions. Thus, owing to the large amount of endogenous metal ions present in tumor cells, side reactions or the click chemistry reaction can occur in these cells without exogenous copper catalysis (see Supplementary Fig. S1). Our results show that the alkyne reacts with the thiol residue of cysteine or the thiol residue of cysteine on the protein surface by the addition reaction (Fig. 1a). Therefore, the captured protein may not be the true target and instead may be a protein with a cysteine on its surface. In this case, hydrogen peroxide treatment efficiently eliminated the thiol interference (Fig. 1b,c). A lower concentration of hydrogen peroxide protected against thiol interference and other interferences from reduced residues and improved the co-localization efficiency of the click chemistry (Fig. 1b,d,e). An imatinib-specific probe and imatinib’s target CD117 were used to perform co-localization analysis. Our results show that the co-localization efficiency increased upon addition of the hydrogen peroxide shield to prevent interference (Fig. 1f).

**Figure 2:**
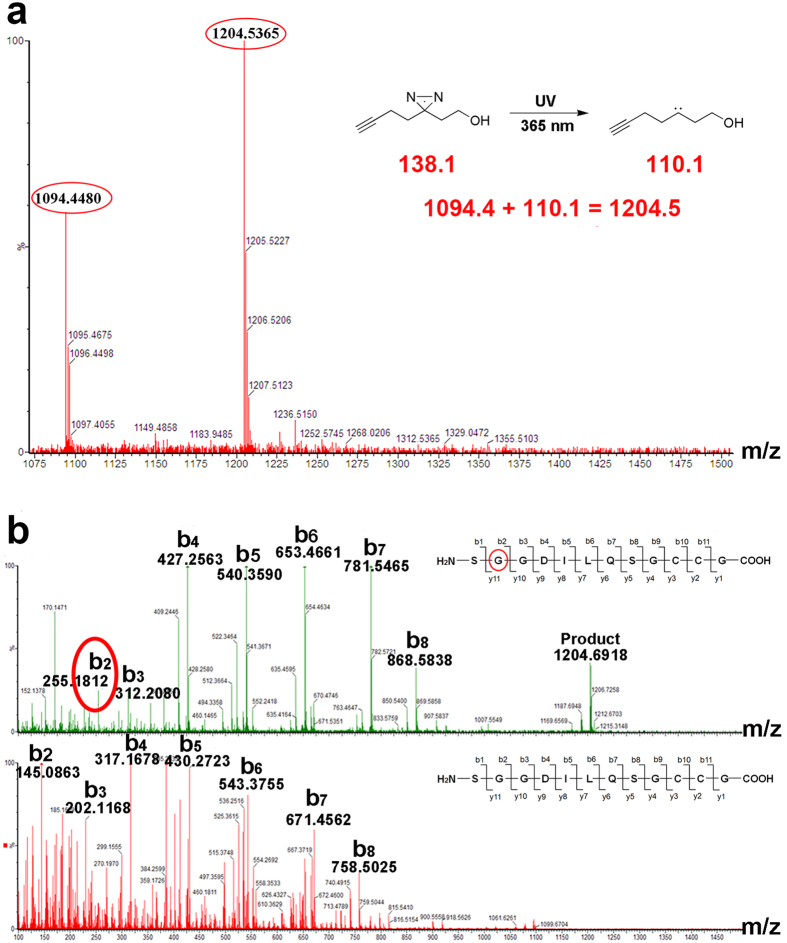
Mass spectrometry of peptide P1 (thioredoxin reductase 1) reacting with alkyl diazirine. (**a**) Reaction mechanism of the photo-affinity group and the changes in molecular weight of the reactant and product. (**b**) Mass/mass spectrometry determination of the binding site of P1 and alkyl diazirine. The results indicate that the second amino acid glycine was the combined site.

The click reaction can be used for target identification and for co-localization analysis of the reaction specificity of organic azides and alkynes^9^,^12^. We synthesized TRITC-azide (1) and FITC-alkyne (2) and assessed their co-localization efficiency in living cells (see Supplementary Figs S2 and S3)^13^. After treatment with hydrogen peroxide, Pearson’s correlation increased from 0.610 to 0.890, and Mander’s overlap increased from 0.756 to 0.906. The co-localization efficiency was changed by using different concentrations of hydrogen peroxide (see Supplementary Fig. S3). In addition to thiol interference in the click reaction, the use of photo-affinity groups (e.g., benzophenones and diazirines) causes interference. Supplementary Table S1 shows that the photo-crosslinking activity of benzophenone was much weaker than that of diazirine. The use of benzophenone as a photo-affinity group was not appropriate, whereas diazirine could be effectively used as a photo-affinity group^14^. However, Table S1 also shows that the labeling efficiency of diazirine was strongly dependent on wavelength (see Supplementary Fig. S4). Figure 2a shows that diazirine exhibited the best photo-crosslinking activity under 365 nm UV irradiation^15^,^16^. The binding site of diazirine reacted with peptide 1, as shown in Fig. 2b.

**Figure 3:**
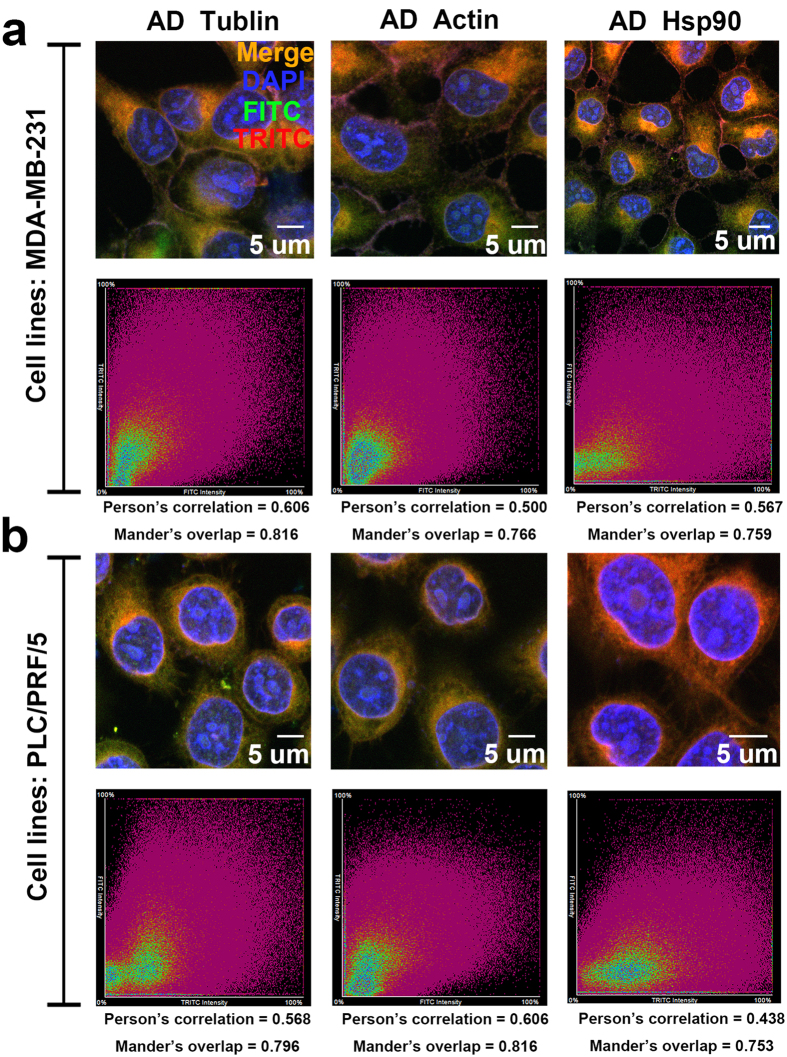
Analysis of the co-localization of alkyl diazirine (AD) and tubulin, actin and HSP90 in 231 and PLC cells, on the basis of immunofluorescence and co-focusing experiments. (**a**) Co-localization of AD and tubulin, actin and HSP90 in 231 cells. (**b**) Co-localization of AD and tubulin, actin and HSP90 in PLC cells.

In living cells, the transport of proteins depends on the cytoskeleton, and the activity and stability of proteins depend on stress proteins (e.g., heat shock proteins)^17^,^18^,^19^,^20^. These assistant proteins reside close to the target protein; thus, they can easily be captured by the photo-affinity groups. In many experiments, we found that tubulin, actin and HSP90 were frequently captured^19^,^21^,^22^. We sought to validate the relationships among these proteins and to determine whether they are bioactive compounds or off-target sites of the photo-affinity groups. We used photo-affinity groups without drug guidance, as well as fluorescence antibody staining to perform co-localization analyses. Figure 3 shows that tubulin, actin and HSP90 reacted with the photo-affinity probes, and the results of co-localization were clear. We also confirmed that the reaction did not vary among different cell lines, as shown in supplementary Figure S5. Therefore, when cytoskeleton proteins or stress proteins are captured, the interference must be carefully eliminated.

In conclusion, this study systematically analyzed the problems associated with the cellular click reaction and photo-affinity groups and revealed that improvements can be made by focusing on the following three points. First, cells can be pretreated with a low concentration of hydrogen peroxide to shield against thiol interference. Second, the use of benzophenone as a photo-affinity group is not appropriate, and although diazirine is effective, it may result in a higher rate of false positives among the identified targets. Furthermore, activation of diazirine strongly depends on wavelength. Finally, cytoskeleton proteins and stress proteins often give rise to interference and should be carefully eliminated. The specificity of target identification can be improved by the optimization of these three points. In the present study, we discuss several limitations of the use of the click reaction in living cells and describe important supplementary techniques for photo-affinity probes based on the click reaction.

## Methods

### Cell culture

Human carcinoma cell lines MDA-MB-231, NIH-3T3 and PLC-PRF-5 were obtained from KeyGen Biotech (Nanjing, China). The cells were grown in RPMI-1640 medium (Hyclone; GE Healthcare, Connecticut, US) and were supplemented with 10% fetal bovine serum (FBS) (Hyclone) and antibiotics (50 units/mL penicillin and 50 μg/mL streptomycin). All cultures were maintained at 37 °C in a humidified atmosphere containing 5% CO_2_.

### Synthesis of FITC-alkyne

Under an argon atmosphere, triethylamine (100.0 μL, 717 μmol) and FITC (commercial product; 90%, HPLC; 143 mg, 330 μmol) were successively added to a solution of 2-Propynylamine (100 mg, ~1.82 mmol) in dichloromethane (5 mL) at room temperature. After stirring for 24 hours at the same temperature, the reaction solution was concentrated under reduced pressure using an evaporator. The residue was purified by flash column chromatography (dichloromethane/methanol = 9/1) to yield FITC-alkyne (110 mg, 226 μmol) as an orange solid. ^1^H NMR (400 MHz, DMSO-d_6_) δ 3.09 (t, J = 2.45 Hz, 1H), 3.90 (d, J = 2.45 Hz, 2H), 6.54 (dd, 2H, J = 2.2, 8.6 Hz), 6.61 (d, 2H, J = 8.6 Hz), 6.65 (d, 2H, J = 2.2 Hz), 7.18 (d, 1H, J = 8.4 Hz), 7.72 (dd, 1H, J = 1.7, 8.4 Hz), 8.21 (d, 1H, J = 1.7 Hz), 8.25–8.40 (br, 1H), 9.80–10.60 (br, 1H). Rhodamine-azide (TRITC-azide) was synthesized as previously described^13^.

### Toxicity assay of the fluorescence probe

Human carcinoma cell lines MDA-MB-231, NIH-3T3 and PLC-PRF-5 were used in the assay according to the same methods. The cells were cultured in 96-well plates, Varying concentrations of the fluorescence probe (FITC-alkyne and TRITC-azide) were added to the experimental groups (100 μM, 50 μM, 25 μM, 12.5 μM, or 6.25 μM); DMSO (final concentration of 1%) was used as the negative control. Cell morphology and growth were used to determine the levels of toxicity. All of the fluorescent probes were deemed safe, and no cytotoxicity was observed.

### Fluorescent probe localization in living cells with or without hydrogen peroxide treatment

The human carcinoma cell lines MDA-MB-231 and PLC-PRF-5 were cultured in a 24-well plate. The FITC-alkyne fluorescent probe (20 μM) or TRITC-azide fluorescent probe (10 μM) was added to the different negative control groups. After 4 hours, the other fluorescent probes were added to the corresponding groups. Catalytic materials (copper sulfate solvent: 1 mM; TCEP: 1 mM; TBTA: 100 μM) were added to the experimental groups in the second addition together with the fluorescence probe. After 4 hours, the medium was changed to the normal culture medium and the cells were incubated for several hours. Finally, the cells were stained with DAPI (blue) and mounted and viewed using a laser scanning confocal microscope (Nikon, Japan). The cells were washed three times with PBS before each new operation described above. In the case of hydrogen peroxide treatment, different concentrations of hydrogen peroxide (0.3%, 0.1%) were added before the fluorescent probe for a reaction time of 1 minute.

### Immunofluorescence staining

Human carcinoma cell lines MDA-MB-231 and PLC-PRF-5 were used in this assay. The diazirine-alkyne solution (100 nm) was added to the experimental group. After incubation for 10 hours, the cells were irradiated for 30 minutes under 365 nm UV light. The current medium was then replaced with normal culture medium. Adherent cells were fixed with pre-cooled methanol for 5 minutes. Click reaction solvents (copper sulfate solvent: 1 mM; TCEP: 1 mM; TBTA: 100 μM; TRITC-azide: 20 μM) were added and incubated for 1 hour. The cells were coated with 5% FBS (0.1% triton) for 30 minutes, and the primary antibodies against tubulin, actin (FITC), and Hsp90β (1:200 dilution) were then added and incubated overnight at 4 °C. The secondary antibodies (1:200 dilution) against tubulin (FITC) and HSP90β (FITC) were added and incubated for 40 minutes at room temperature in the dark. Finally, the cells were stained with DAPI (blue), mounted and viewed using a laser scanning confocal microscope (Nikon, Japan). The cells were washed with PBS three times before each new operation described above.

### Activity test of diazirine and benzophenone

The four peptides were obtained from the GenScript Corporation (Nanjing, China). The synthesized probes and peptides were incubated for 1 hour at 37 °C, and after the incubation, the complex was irradiated for 1 hour under 365 nm UV light. Then, mass spectrometry was used to detect the reactants and products, and the peak area was selected as a relative quantitative value.

## Additional Information

**How to cite this article**: Zhong, W. *et al*. Problems and Solutions in Click Chemistry Applied to Drug Probes. *Sci. Rep.* **6**, 35579; doi: 10.1038/srep35579 (2016).

## Supplementary Material

Supplementary Information
