# Supplementary material for: Problems and Solutions in Click Chemistry Applied to Drug Probes
Source: Sci Rep. 2016 Oct 26;6:35579. doi: 10.1038/srep35579 (PMC5080546; doi:10.1038/srep35579)
Supplement: Supplementary Information [file srep35579-s1.pdf]

Supplemental Information

Problems and Solutions in Click Chemistry Applied to Drug Probes

Weilong Zhong, [1,2,#] Bo Sun, [1,2,#] Cheng Lu, [1,2#] Hengheng Yu, [1,2,#] Changhua Wang, [1,2] Lingfei He, [1,2] Ju Gu, [1,2] Shuang Chen, [2] Yanrong Liu,[2] Xiangyan Jing,[1,2] Zhun Bi,[1] Guang Yang,[1] Honggang Zhou, [1,2] Tao Sun,[1,2,*] Cheng Yang[1,2,*]

[1]State Key Laboratory of Medicinal Chemical Biology and College of Pharmacy, Nankai University, Tianjin, China

[2]Tianjin Key Laboratory of Molecular Drug Research, Tianjin International Joint Academy of Biomedicine, Tianjin, China

*Correspondence:

Cheng Yang: cyang66_2001@yahoo.com;

Tao Sun: sunrockmia@hotmail.com

# These authors contributed equally to this work.

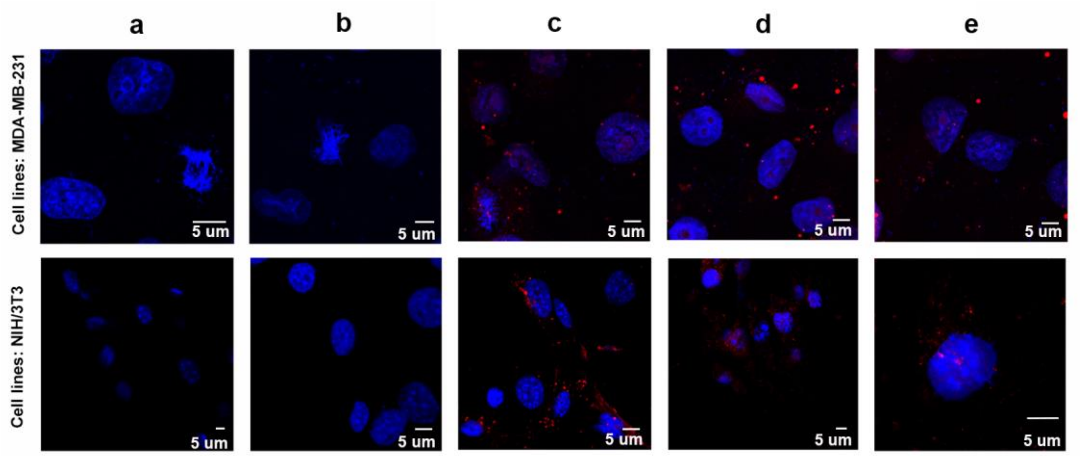

Figure S1. Related to Figure 1. The click reaction results in catalyzed copper ions with or without iqwww copper ion catalysis. Confocal fluorescence results for **a**, rhodamine. **b**, rhodamine-piperazine. **c**, rhodamine-piperazine-alkyne. **d**, rhodamine-piperazine-alkyne treatment with EDTA. **e**, rhodamine-piperazine-alkyne without catalysts.

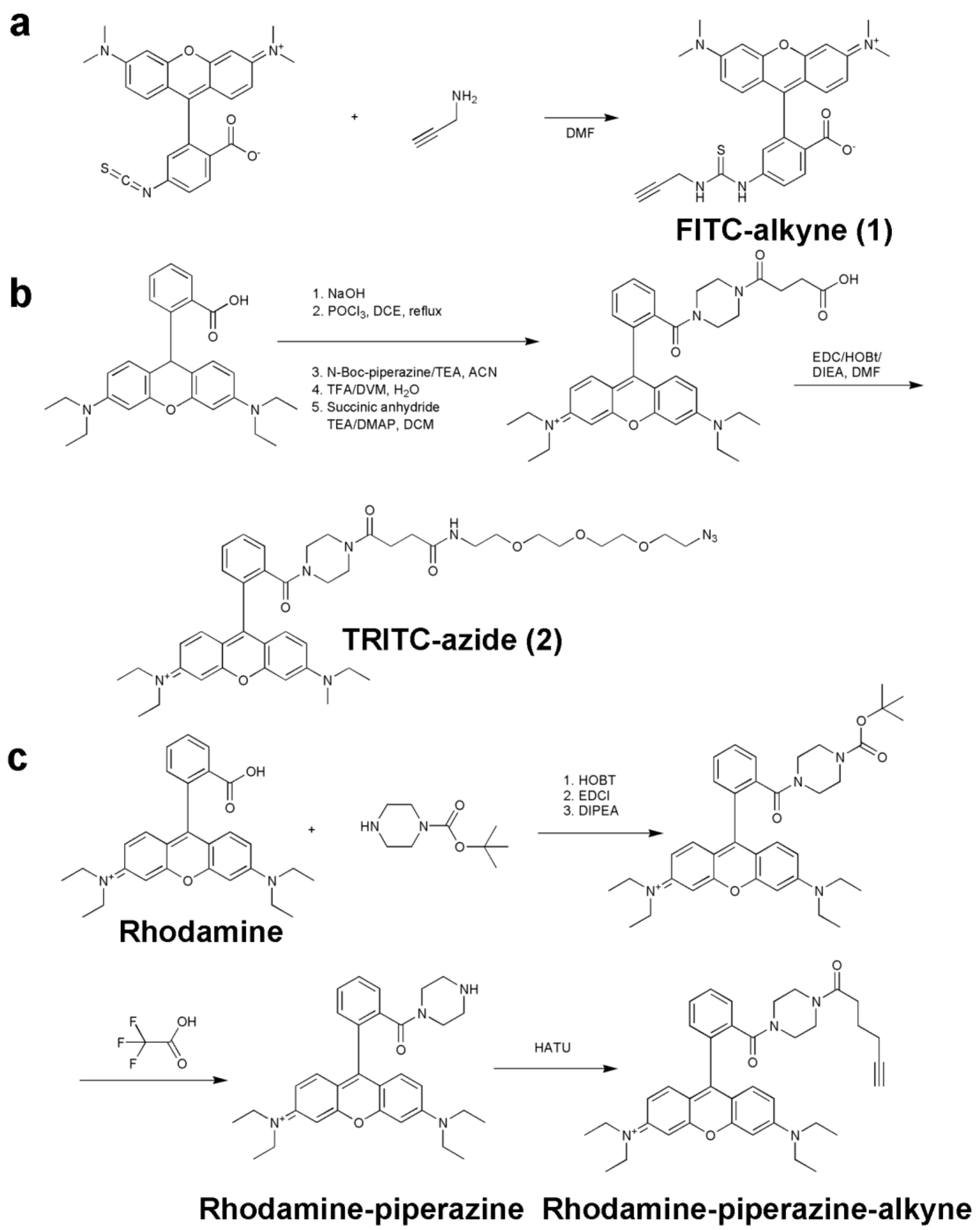

Figure S2. Related to Figure 1. Synthesis of FITC-alkyne, TRITC-azide and rhodamine-piperazine-alkyne. **a**, Synthesis of FITC-alkyl. **b**, Synthesis of TRITC-azide. **c**, Synthesis of rhodamine-piperazine-alkyne.

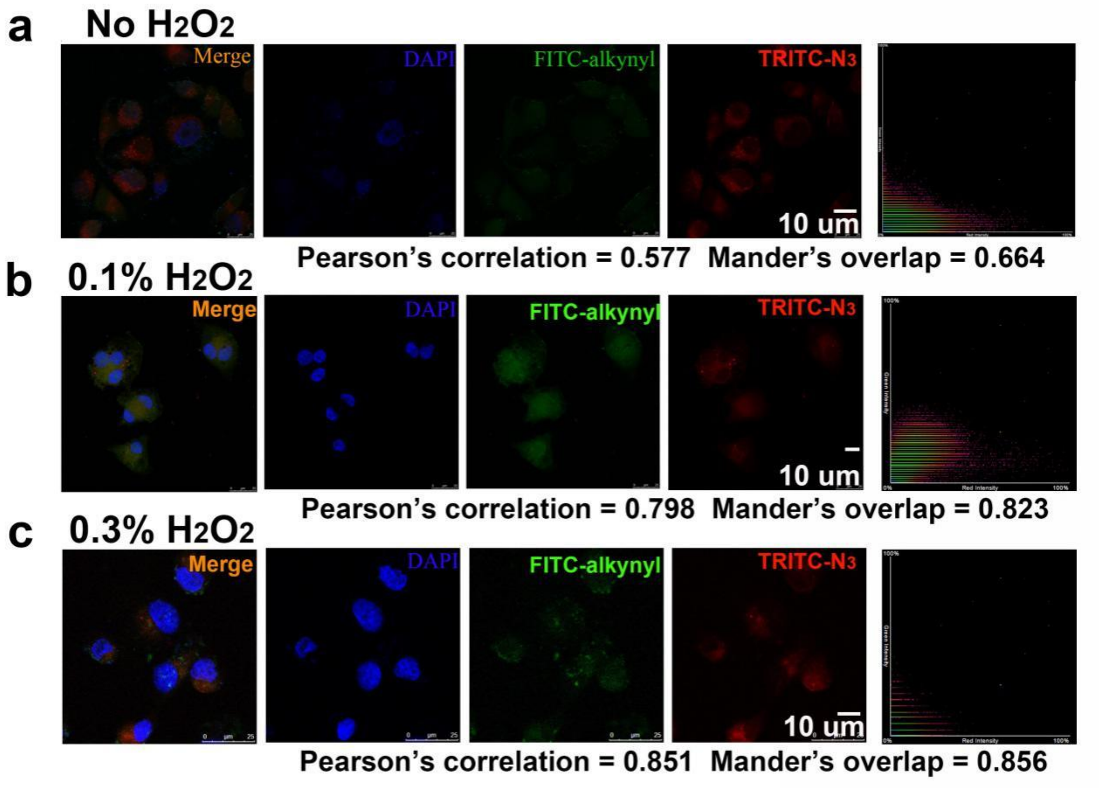

Figure S3. Related to Figure 1. Co-localization efficiency at different hydrogen peroxide concentrations. **a**, Cells without hydrogen peroxide treatment. Effects of **b**, 0.1% hydrogen peroxide treatment and **c**, 0.3% hydrogen peroxide treatment. Hydrogen peroxide eliminates the interference of cysteine and improves the co-localization efficiency of FITC-alkyne and TRITC-azide in living cells.

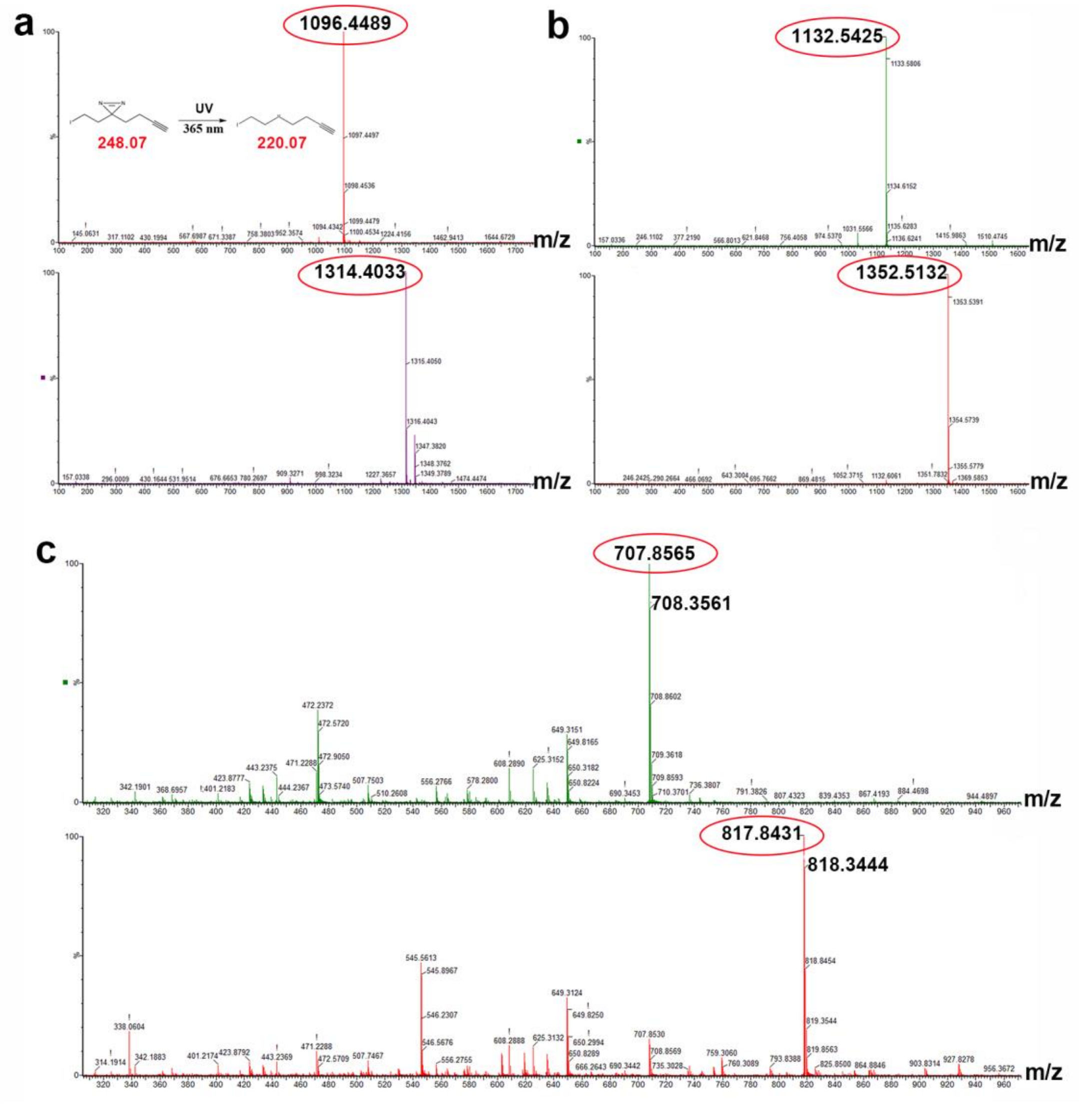

Figure S4. Related to Figure 2. Molecular weight changes of peptide 1-3 after reaction with alkyl diazirine under 365 nm UV irradiation.

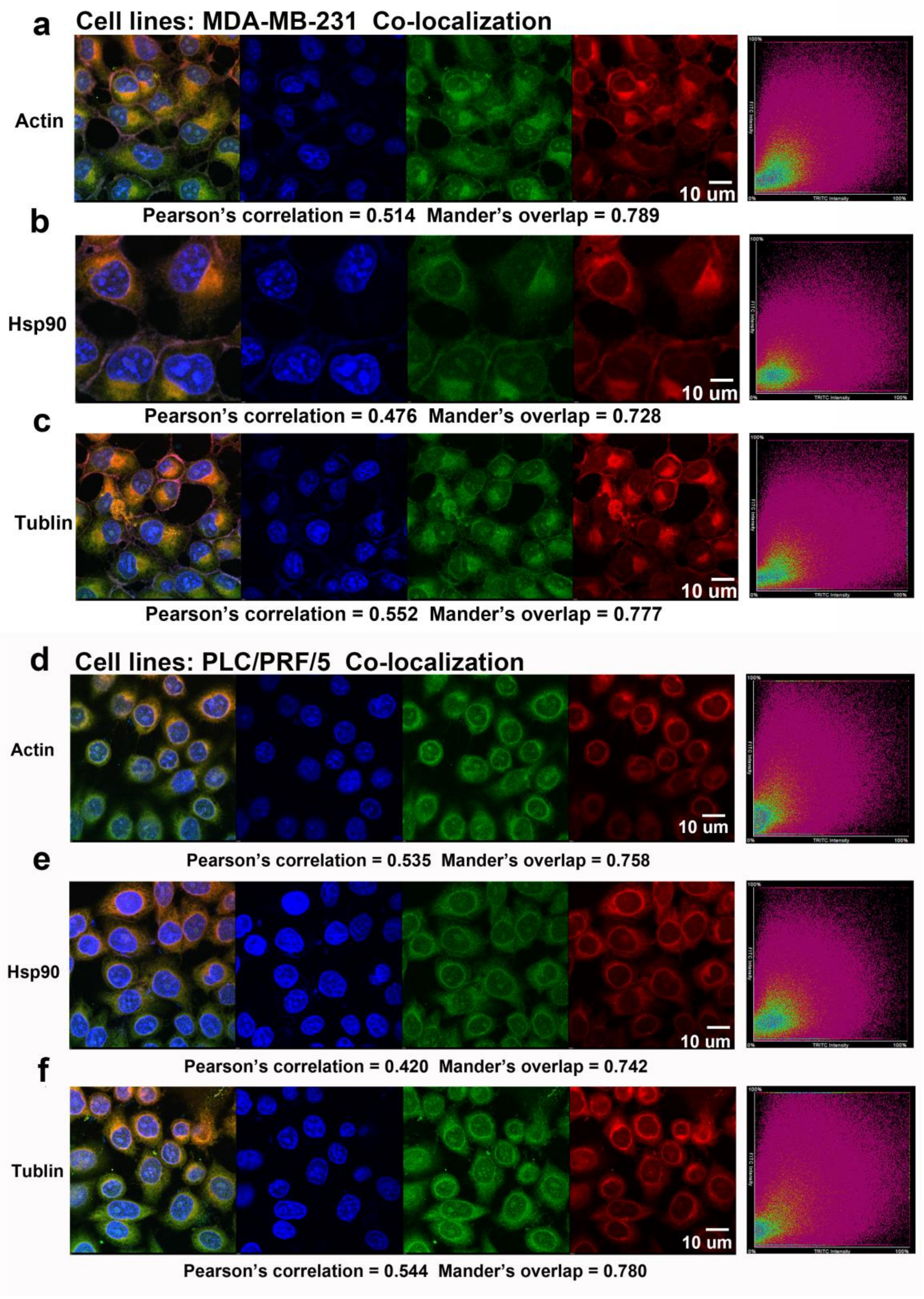

Figure S5. Related to Figure 3. Co-localization of alkyl diazirine (AD) and tubulin, actin and HSP90 in 231 and PLC cells.

Table S1. The reaction efficiencies of four peptides reacting with benzophenone and alkyl diazirine (AD).

	365 nm				350 nm			
Peptide	P1	P2	P3	P4	P1	P2	P3	P4
Benzophenone	0.539<br>275.899	0.444<br>919.674	1.943<br>177.186	1.965<br>26.195	NA	NA	NA	15.403<br>1366.7
Alkyl diazirine	2326.055<br>1573.198	2532.69<br>3840.942	2746.684<br>1497.542	2.891<br>261.512	NA	121.66<br>3474.202	70.738<br>6637.455	NA

Note:

P1: SGGDILQSGCCG; P2: INTQWLLSGT; P3: ARNEHKMFPWV; P4: ARDCEHKMFPYV.

Red values represent the peak area of the products, and blue values represent the peak area of the reactants. The results show that alkyl diazirine readily reacts with the peptide under 365 nm UV irradiation. In contrast, the benzophenone reaction efficiency was low under both 350 nm and 365 nm UV irradiation.
